# Association between serum neutrophil extracellular traps and carotid intima-media thickness in type 2 diabetes: a cross-sectional study

**DOI:** 10.3389/fendo.2026.1769035

**Published:** 2026-03-17

**Authors:** Xinyan Jin, Shanshan Yu, Cheng Chen, Wenjun Sha, Tao Lei

**Affiliations:** Department of Endocrinology, Putuo Hospital, Shanghai University of Traditional Chinese Medicine, Shanghai, China

**Keywords:** carotid atherosclerosis, carotid intima-media thickness, diabetic vascular complications, neutrophil extracellular traps, type 2 diabetes mellitus

## Abstract

**Background:**

Atherosclerosis is a chronic low-grade inflammatory vascular disease and serves as the core pathological basis for cardiovascular complications in patients with type 2 diabetes mellitus (T2DM). Upon activation, neutrophils release structures known as neutrophil extracellular traps (NETs), which exhibit pro-inflammatory properties and contribute substantially to atherosclerotic progression. Nevertheless, the precise relationship linking NETs to carotid atherosclerosis (CAS) among individuals with T2DM has not been fully elucidated. The objective of this research is to examine the connection between circulating NETs concentrations and carotid intima-media thickness (CIMT) values.

**Methods:**

This study included 356 patients diagnosed with T2DM who were hospitalized in the Department of Endocrinology at Shanghai Putuo District Central Hospital between January 2024 and June 2025. Participants were stratified into three subgroups according to CIMT values. Serum NETs content was measured with a Human Neutrophil Extracellular Traps Enzyme-Linked Immunosorbent Assay Kit. Immunofluorescence staining was applied to evaluate and compare the expression of NETs-related markers across the three CIMT-based groups. Differences in baseline demographic features, laboratory indices, and CIMT measurements among the groups were systematically compared. Spearman rank correlation analysis was performed to explore associations between NETs and relevant clinical or biochemical variables. Factors contributing to CAS were further identified using binary logistic regression analysis. The predictive value of circulating NETs for CAS was assessed by receiver operating characteristic (ROC) curve analysis.

**Results:**

Compared with the CIMT < 1.0 mm group, the 1 ≤ CIMT < 1.5 mm group showed significant increases in body mass index (BMI), diabetes duration, systolic blood pressure (SBP), fasting plasma glucose (FPG), 2-hour postprandial glucose (2hPG), glycated hemoglobin (HbA1c), glycated albumin (GA), total cholesterol (TC), low-density lipoprotein cholesterol (LDL-C), blood urea nitrogen (BUN), and NETs (*P<0.05*). The CIMT ≥ 1.5 mm group exhibited significant increases in BMI, number of smokers, diabetes duration, cumulative advanced glycation end products (AGEs), SBP, diastolic blood pressure (DBP), FPG, 2hPG, HbA1c, GA, TC, LDL-C, serum creatinine (Scr), BUN, estimated glomerular filtration rate (eGFR), urinary albumin-to-creatinine ratio (UACR), and NETs levels (*P<0.05*). Compared with the 1 ≤ CIMT < 1.5 mm group, the CIMT ≥ 1.5 mm group had significantly higher levels of BMI, number of smokers, diabetes duration, cumulative FPG, AGEs, DBP, HbA1c, GA, TC, NETs,eGFR, UACR, and LDL-C (*P<0.05*). Immunofluorescence indicated that with the increasing CIMT grade, the level of spontaneous NET formation by neutrophils in patients showed a significant increase. Spearman correlation analysis revealed that serum NETs levels were positively correlated with CIMT (*r = 0.637*, P<0.001). Logistic regression analysis identified NETs were a risk factor for CAS in T2DM patients (*OR = 1.76, 95%CI 1.40–2.21, P < 0.001*). Analysis using the receiver operating characteristic framework demonstrated that circulating NETs possess substantial diagnostic value for identifying carotid atherosclerosis in patients, as reflected by an area under the curve of 0.877.

**Conclusion:**

Serum NETs levels were independently associated with the presence of CAS in patients with T2DM and exhibited a certain predictive value for identifying CAS. However, due to the limitations of the cross-sectional study design, the causal relationship between the two requires further validation in prospective cohort studies.

## 1 Introduction

Type 2 diabetes mellitus (T2DM) and its associated atherosclerotic cardiovascular diseases constitute a major global public health challenge. Premature death is associated with microvascular and macrovascular complications in 50% of these T2DM patients (1). Among various vascular complications, carotid atherosclerosis (CAS), serving as a window to systemic atherosclerosis, is closely associated with stroke risk (2). Atherosclerosis is thought to be a process driven primarily by elevated levels of low-density lipoprotein cholesterol (LDL-C) and its retention within the vessel wall. While lipid retention remains a major contributor to atherosclerosis, studies have increasingly identified atherosclerosis as an inflammatory process. Involved innate and adaptive immune cells as well as inflammatory mediators, such as cytokines, chemokines, and extracellular vesicles (3). Activated immune cells (eg, T cells, mast cells) and their release of a variety of inflammatory factors (eg, tumor necrosis factor-α, TNF-α; interleukin-1β, IL-1β; interleukin-6, IL-6). These factors not only continuously exacerbate the local inflammatory response, promote smooth muscle cell proliferation and migration, but also disrupt the stability of plaques, resulting in their susceptibility to rupture and triggering acute clinical events. Thus, a chronic, low-grade inflammatory state is a critical link that runs through the development, progression, and ultimately complications of atherosclerosis.

The contribution of innate immunity to the pathogenesis of atherosclerosis has garnered growing research interest in recent years. Neutrophils, as the first line of defense against infection, have a newly discovered immune defense mechanism known as neutrophil extracellular traps (NETs), providing a fresh perspective for understanding chronic inflammatory diseases.NETs are web-like structures released by activated neutrophils, composed of decondensed chromatin DNA scaffolds embedded with histones and various granular proteases, such as myeloperoxidase and neutrophil elastase (4, 5). This mechanism is originally designed to physically capture and efficiently eliminate pathogens. However, excessive formation or inadequate physiological clearance can result in NETs components being exposed in the vascular lumen, which then attack the body’s own tissues, acting as a ‘driver’ of inflammation and tissue damage (6). Studies have shown that NET components possess direct cytotoxic effects, capable of inducing endothelial cell apoptosis and pyroptosis (7). For example, its core component, extracellular histones, can directly disrupt endothelial cell membranes and activate Toll-like receptors, triggering intense inflammatory responses. Meanwhile, MPO catalyzes the production of the potent oxidant hypochlorous acid, exacerbating oxidative stress and impairing vascular function. Furthermore, NETs can act as autoantigens, amplifying local inflammatory cascades and promoting thrombosis, thereby playing a key role in the initiation, progression, and instability of atherosclerotic plaques (8).Importantly, metabolic stress factors characteristic of T2DM disease, such as high glucose, free fatty acids, and advanced glycation end products (AGEs), have been demonstrated to be potent inducers of NETs formation (8–10). Specifically, these products can directly “educate” and hyperactivate neutrophils through multiple pathways such as activating protein kinase C and increasing reactive oxygen species generation, making them more prone to undergo NETosis. This process thereby converts general metabolic dysregulation into a specific and destructive innate immune response.This suggests that NETs may represent a critical pathological bridge linking diabetic metabolic disturbances to vascular inflammatory damage.

However, there are few reports of serum NETs levels measuring the severity of carotid atherosclerosis in people with type 2 diabetes. Therefore, the aim of this study was to verify the correlation between serum NETs levels and carotid intimal thickness. In order to provide new evidence for revealing the inflammatory mechanism of diabetic vascular complications and provide a theoretical basis for finding potential intervention targets.

## 2 Materials and methods

### 2.1 Study participants

This study was conducted in accordance with the principles of the Declaration of Helsinki. A total of 356 patients with type 2 diabetes mellitus (T2DM) admitted to the Department of Endocrinology of Shanghai Putuo District Central Hospital between January 2024 and June 2025 were selected for this study. The preliminary inclusion criteria are as follows: patients who meet the American Diabetes Association (2019) T2DM standards (11), aged between 30 and 75 years. Exclusion criteria included: Patients with T1DM and other types of DM; patients with acute cardiovascular and cerebrovascular diseases; patients with severe infections or trauma; patients with severe liver and kidney dysfunction; patients with hematological diseases, acute infections, or malignant tumors; Patients with autoimmune diseases such as Sjögren’s syndrome; pregnant and breastfeeding women; patients taking statins, fibrates, or antiplatelet aggregation drugs. Patients with regular use of nonsteroidal anti-inflammatory drugs (NSAIDs) or corticosteroids within 4 weeks prior to enrollment were also excluded.According to the report of the American College of Cardiology/American Heart Association Clinical Practice Guidelines Work Group (12), patients were divided into three groups according to carotid intima-media thickness (CIMT) levels: CIMT < 1.0 mm, 1 ≤ CIMT < 1.5 mm, and CIMT ≥ 1.5 mm. This study was approved by the Ethics Committee of Putuo District Central Hospital, Shanghai (Ethics Approval No.: PTEC-A-2025-5-1), and informed consent was obtained from each participant prior to the examination.

### 2.2 Research methods

#### 2.2.1 Blood sample collection and processing

All participants fasted for over 8 hours, and fasting venous blood was collected from the antecubital vein in the morning.A portion of the samples is utilized for immediate biochemical analysis, while the serum and plasma obtained from the remaining portion after centrifugation are preserved at -80 °C. Fasting blood glucose (FPG), total cholesterol (TC), triacylglycerol (TG), high-density lipoprotein cholesterol (HDL-C), low-density lipoprotein cholesterol (LDL-C), serum uric acid (SUA), and serum creatinine (Scr) were measured using the Roche Diagnostics cobas8000 fully automated biochemical analyzer. Glycated hemoglobin (HbA1c) was detected by high-performance liquid chromatography (using the U.S. BIO-RAD D10 hemoglobin A1c analyzer). Urinary albumin and urinary creatinine were first determined by immunoradiometric assay, and their ratio was then calculated to obtain the urinary albumin-to-creatinine ratio (UACR). Serum NETs levels were measured using a Human Neutrophil Extracellular Traps ELISA Kit (purchased from Jianglai Biotechnology, JL47452, China).

#### 2.2.2 CIMT measurement

Carotid ultrasound examination is performed using a Phillips EPIQ color Doppler ultrasound diagnostic instrument with a frequency of 7.0–10 MHz. The ultrasound assessments were performed independently by two certified sonographers in accordance with a standardized imaging protocol (13). The sonographers responsible for CIMT measurements had no access to participants’ clinical diagnostic information, laboratory test results, or study groupings based on CIMT values.All participants were in the supine position, with their heads slightly turned to the opposite side of the examination, fully exposing the neck. B-mode ultrasound probe was used to measure the vertical distance between the luminal surface of the intima and the outer surface of the media at the distal common carotid artery 1.0–1.5 cm proximal to the carotid bifurcation and/or at the origin of the internal carotid artery. In cases where plaques were present, plaque size was also measured. The average CIMT value from both sides was calculated. To evaluate the reliability of CIMT measurements, we calculated the intraclass correlation coefficient (ICC) for the measurements obtained by two independent sonographers.

#### 2.2.3 Measurement of AGEs

The accumulation of advanced glycation end products in tissue was assessed in all participants using an AGE Pro non-invasive detector (Anhui Yikangda Optoelectronic Technology Co., Ltd., model AP20210214). Measurements were performed in a dark room. After resting for 15 minutes, each subject placed the cleansed skin of the volar side (palmar side) of the forearm under the probe. The device automatically emitted ultraviolet light with a wavelength range of 300–420 nm and detected the emitted fluorescence within the 420–600 nm wavelength range. Simultaneously, the skin reflection spectrum was measured to correct for the influence of skin pigmentation. The final result was expressed as the skin autofluorescence value.

#### 2.2.4 Isolation of human neutrophils and immunofluorescence staining of NETs

Peripheral blood samples were collected from three groups of patients (10 cases per group, 10 mL of blood per case). Neutrophils were isolated from human peripheral blood using a commercial kit (LZS11131, TBD Sciences, Tianjin, China). Cell viability was determined by the trypan blue exclusion method and ranged from 95% to 97%. Isolated neutrophils (1×10^6^ cells) were cultured in RPMI-1640 medium (11875093, Gibco, USA) supplemented with 10% fetal bovine serum. After 4 h of cell adherence, cells were permeabilized with Triton X-100 (HM0919Y, Yifan Biotechnology) for 10 min, fixed with 4% paraformaldehyde for 30 min, and blocked with 5% bovine serum albumin (4240GR500, BioFroxx) at room temperature for 1 h. Two independent single-antigen immunofluorescence staining assays were performed, with each antigen processed in separate wells in parallel. (a) MPO staining only.After blocking, cells were incubated with primary antibody against myeloperoxidase (MPO, 1:100, #14569, Cell Signaling Technology, USA) overnight at 4 °C. After incubation, the primary antibody was removed and cells were washed three times with PBS. Corresponding secondary antibody Alexa Fluor 488-conjugated goat anti-rabbit IgG (GB25303, Servicebio, China) was added and incubated for 1 h at room temperature in the dark.After three PBS washes, cells were stained with DAPI solution (C1002, Beyotime, China) for 10 min at room temperature in the dark to label nuclei.After a final three washes with PBS, cells were mounted with anti-fluorescence quenching mounting medium and imaged under an inverted fluorescence microscope. (b) NE staining only. Another set of adherent neutrophils was subjected to an identical procedure. Cells were incubated with primary antibody against neutrophil elastase (NE, 1:400, #89241, Cell Signaling Technology, USA) overnight at 4 °C. After incubation, the primary antibody was removed and cells were washed three times with PBS.Cy3-conjugated goat anti-rabbit IgG (GB21303, Servicebio, China) was added and incubated for 1 h at room temperature in the dark.

Subsequent DAPI staining, mounting, and imaging were performed identically to the MPO staining group.MPO and NE were never stained in the same sample; each marker was processed in parallel in separate wells.In addition, negative control staining was performed to verify staining specificity: primary antibody was replaced with blocking solution, and only secondary antibody was applied. Negative controls showed extremely low background fluorescence, excluding non-specific background interference.

### 2.3 Statistical analysis

Statistical analysis was performed using SPSS 27.0 software, and figures were generated with GraphPad Prism 9.4.1. The distribution of all continuous variables was assessed using the Kolmogorov-Smirnov test. Normally distributed continuous variables are expressed as mean ± standard deviation ( $\bar{\text{x}}$ ± s). Non-normally distributed continuous variables are expressed as median and interquartile range (IQR, Q1-Q3), and intergroup comparisons were conducted using nonparametric tests. Categorical variables are reported as frequency (n) and percentage (%), and the chi-square test was applied. The relationship between NETs and CIMT was evaluated using Spearman correlation and binary logistic regression analysis. Receiver operating characteristic (ROC) curves were constructed to assess the sensitivity and specificity of NETs in predicting CAS. A *P-value < 0.05* was considered statistically significant.

## 3 Results

### 3.1 Basic characteristics of the study population

We first evaluated the interobserver reliability of ultrasound measurements using the ICC. The ICC for single measurements was 0.761 (95% CI: 0.580–0.871, P < 0.001), and the ICC for average measurements was 0.865 (95% CI: 0.734–0.931, P < 0.001), indicating good measurement consistency between the two examiners. The main demographic characteristics of the study group are presented in **Table 1**. Based on CIMT levels, participants were categorized into three groups: CIMT < 1.0 mm (n = 113), 1.0 mm ≤ CIMT < 1.5 mm (n = 146), and CIMT ≥ 1.5 mm (n = 97).Compared to the CIMT < 1.0 mm group, the 1.0 mm ≤ CIMT < 1.5 mm group exhibited significant increases in BMI, diabetes duration, systolic blood pressure (SBP), FPG, 2-hour postprandial glucose (2hPG), HbA1c, glycated albumin (GA), TC, LDL-C, blood urea nitrogen (BUN), NETs, and CIMT (*P < 0.05*).In the CIMT ≥ 1.5 mm group, compared to the CIMT < 1.0 mm group, elevated levels were observed in BMI, number of smokers, diabetes duration, AGEs, SBP, diastolic blood pressure (DBP), FPG, 2hPG, HbA1c, GA, TC, LDL-C, Scr, BUN, estimated glomerular filtration rate (eGFR), UACR, NETs, and CIMT (*P < 0.05*). Furthermore, compared to the 1.0 mm ≤ CIMT < 1.5 mm group, the CIMT ≥ 1.5 mm group showed higher levels of BMI, number of smokers, diabetes duration, AGEs, DBP, FPG, HbA1c, GA, TC, LDL-C, eGFR, UACR, NETs, and CIMT (*P < 0.05*). No significant differences were observed among the three groups in terms of number of alcohol drinkers, ejection fraction, HDL-C, TG, serum uric acid(SUA), and liver function including aspartate aminotransferase (AST), alanine aminotransferase (ALT), and gamma-glutamyl transferase (GGT) (*P > 0.05*).

**Table 1:** Comparison of baseline characteristics among patients with different degrees of CAS.

Variables	CIMT≥1.5	1≤CIMT<1.5	CIMT<1	*χ^2^*	*P value*
Participants	97	146	113		
Sex(male)	54/97 (55.7%)	77/146 (52.7%)	71/113 (68.2%)	2.71	0.259
Age(years)	59(53,65)	60(51,67.75)	61(56,66)	4.66	0.097
BMI(kg/m^2^)	25.28(23.29,28.04)	24.16(21.88,25.66)	24.11(22.39,25.91)	13.66	0.01
SBP(mmHg)	130(125.00,140.00)	130(124.00,138.00)	128(124.00,135.50)	7.94	0.019
DBP(mmHg)	86(80.05,89.00)	80(75.00,84.00)	79(74.00,86.50)	42.68	<0.001
Tobacco Use History(%)	32/97 (33%)	24/146 (16.6%)	17/113 (15%)	12.71	0.02
Alcohol Use History(%)	24/97 (24.7%)	27/146 (21.3%)	25/113 (22.1%)	1.415	0.493
Duration of DM	10(5.50,16.00)	8(3.00,10.25)	5(2.00,10.00)	38.433	<0.001
Hypertension(%)	53/97 (54.6%)	81/146 (55.5%)	77/113 (68.1%)	5.415	0.067
Ejection Fraction(%)	64(60,67)	63(60,66)	62(60,66)	3.08	0.215
AGEs	98.20(88.60,107.10)	89.90(84.65,101.78)	89.80(83.70,97.60)	18.30	<0.001
FPG(mmol/L)	9.40(7.86,12.18)	8.35(6.94,9.97)	6.56(5.48,8.01)	59.30	<0.001
2hPG(mmol/L)	14.50(11.03,16.35)	12,80(10.77,15.57)	10.16(7.99,12.61)	40.64	<0.001
HbA1c(%)	11.10(10.00,12.00)	9.00(8.10,9.83)	7.20(6.60,8.50)	153.08	<0.001
GA	30.12(25.20,34.20)	22.30(19.37,26.60)	18.90(16.10,23.05)	107.99	<0.001
TC(mmol/L)	5.36(4.70,6.26)	4.61(3.94,5.23)	4.18(3.28,4.90)	54.21	<0.001
TG(mmol/L)	1.46(1.08,2.41)	1.33(1.06,1.97)	1.35(1.02,2.08)	2.16	0.34
HDL-C(mmol/L)	1.17(0.97,1.36)	1.15(0.95,1.32)	1.15(1.08,1.28)	1.136	0.57
LDL-C(mmol/L)	3.30 (2.76,3.85)	2.83(2.16,3.61)	2.36(1.66,2.77)	77.61	<0.001
ALT(U/L)	18.00(15.00,23.00)	20.35(14.10,27.85)	16.80(13.20,24.90)	2.96	0.23
AST(U/L)	18.00(15.00,23.00)	18.70(14.95,23.30)	18.20(15.35,23.70)	0.01	0.99
GGT(U/L)	23.00(18.00,37.10)	29.40(18.20,42.60)	22.00(17.10,30.75)	5.42	0.07
SUA(μmol/L)	308.00(244.00,373.00)	327.00(267.00,398.00)	320.00(263.00,393.70)	2.50	0.286
Scr(μmol/L)	63.00(55.00,80.40)	62.00(53.00,72.00)	59.00(52.50,67.70)	12.11	0.002
BUN(mmol/L)	6.70(5.70,8.40)	5.95(5.00,7.43)	5.90(4.95,7.50)	13.78	0.001
eGFR (mL/min/1.73 m^2^)	101.71(80.73,107.79)	104.55(89.89,119.24)	106.62(95.46,119.34)	17.01	<0.001
UACR	18.81(6.59,67.92)	10.06(5.27,27.95)	8.31(5.05,27.16)	9.15	0.01
NETs(ng/ml)	19.02(16.30,21.54)	14.98(13.96,16.59)	10.73(8.71,12.87)	165.71	<0.001

NETs levels in three groups: CIMT < 1.0 mm, 1 ≤ CIMT < 1.5 mm, and CIMT ≥ 1.5 mm.

**Figure 1:**
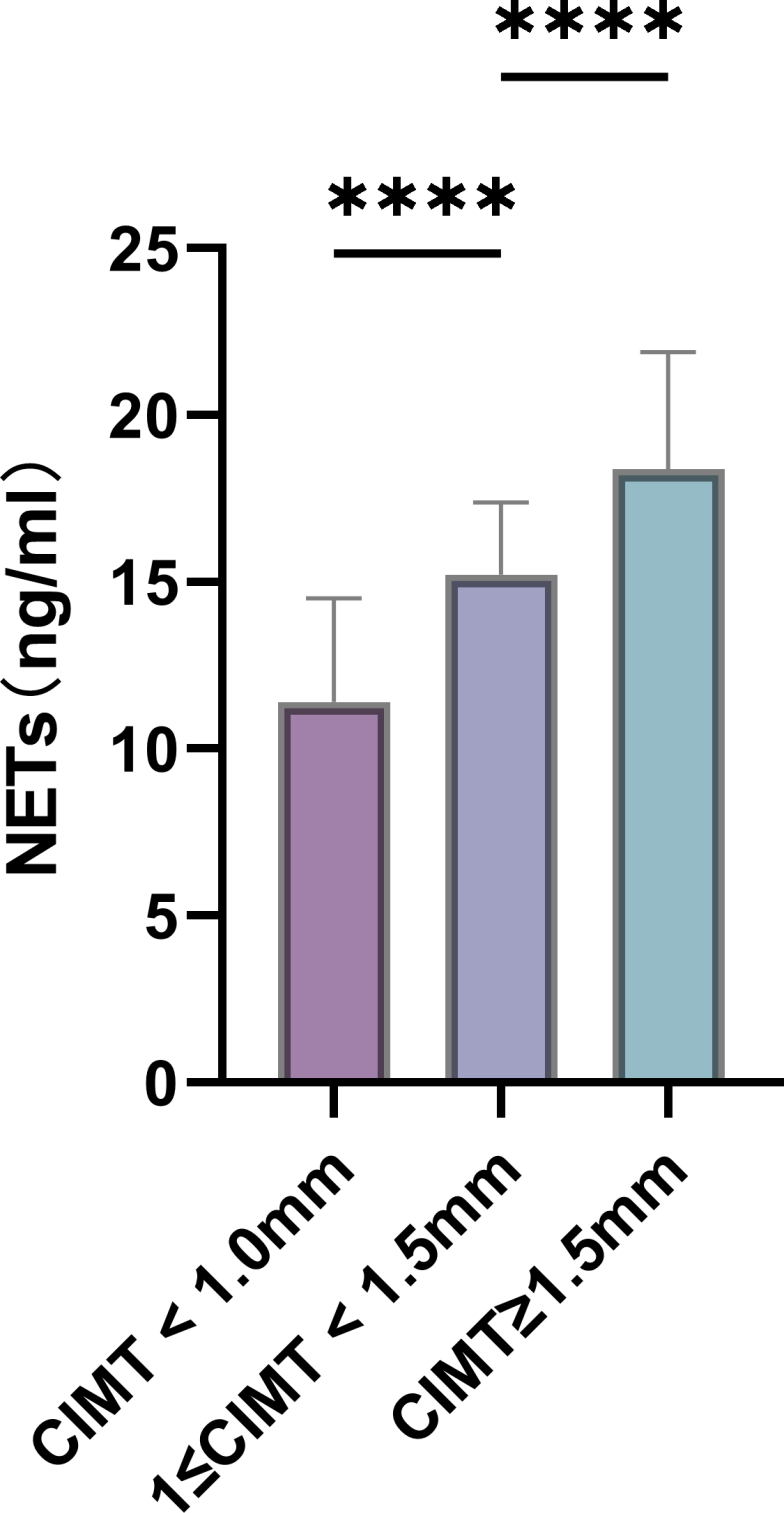
Serum NETs levels in the three groups: CIMT < 1.0 mm, 1 ≤ CIMT < 1.5 mm, and CIMT ≥ 1.5 mm. (****P < 0.0001).

In this study, Elisa method was used to detect the content of NETs in human serum, and the results showed that the content of NETs with 1 ≤ CIMT < 1.5 mm and CIMT ≥ 1.5 mm was significantly increased compared with CIMT < 1.0 mm (P < 0.001). And CIMT ≥ 1.5 mm group was significantly higher and compared with 1 ≤ CIMT < 1.5 mm (P < 0.001). These results suggest that NETs content increases with increasing CIMT values (**Figure 1**).

### 3.2 Comparison of spontaneous NETs of neutrophils in peripheral blood among the three groups

**Figure 2:**
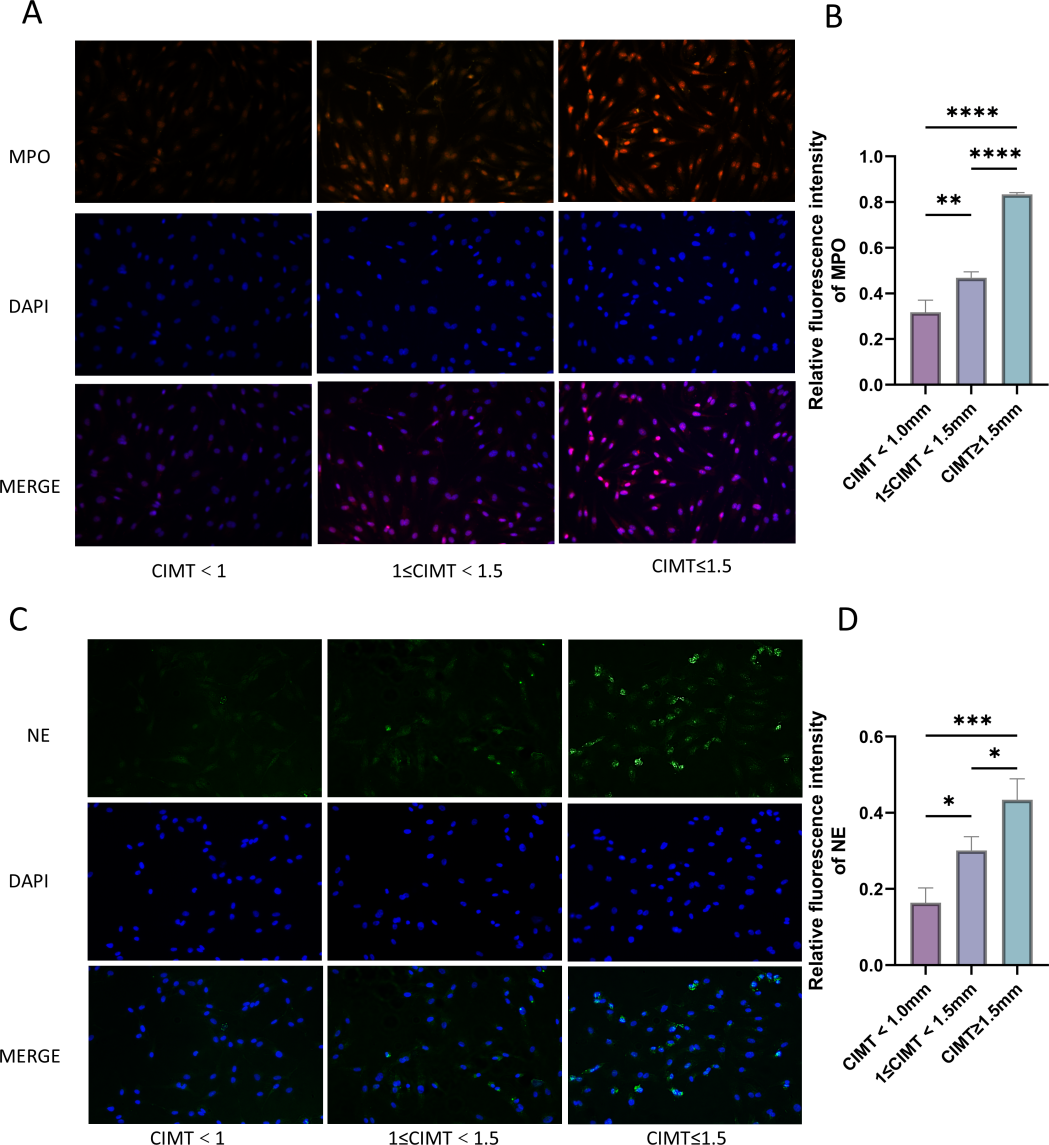
Spontaneous NETs in peripheral blood neutrophils of three groups. **(A, B)** Immunofluorescence detection of MPO levels in the three groups; **(C, D)** Immunofluorescence detection of NE levels in the three groups. (*P < 0.05; **P < 0.01; ***P < 0.001, ****P < 0.0001).

MPO and NE are markers of NETs (14, 15). We detected the content of two markers by immunofluorescence in the three groups of CIMT < 1.0 mm, 1 ≤ CIMT < 1.5 mm, and CIMT ≥ 1.5 mm, and speculated that NETosis occurred in neutrophils in the three groups. The results showed that the mean fluorescence intensity of MPO and NE in the CIMT ≥ 1.5 mm group was greater than that in the CIMT < 1.0 mm and 1 ≤ CIMT < 1.5 mm groups. The mean fluorescence intensity of MPO and NE in the 1 ≤ CIMT < 1.5 mm group was also greater than that in the CIMT < 1.0 mm group, and this result was the same as the Elisa results above (**Figure 2**).

### 3.3 Correlation analysis between serum NETs Levels and various indicators

**Table 2** presents the relationships between serum NET levels and CIMT, as well as other clinical variables, using Spearman correlation analysis. The results showed that serum NETs levels were positively correlated with duration of diabetes, number of smokers, DBP, CIMT, FPG, 2hPG, HbA1c, GA, TC, and LDL-C (all *P* < 0.05).

**Table 2:** Correlation between NETs levels and other clinical indicators.

Variables	Duration of DM	Tobacco use history	DBP	AGEs	CIMT
*r*	0.196	0.107	0.151	0.150	0.637
*p*	<0.001	0.044	0.004	0.005	<0.001

Variables	FPG	2hPG	HbA1c	GA	TC	LDL-C
*r*	0.352	0.385	0.548	0.457	0.244	0.194
*p*	<0.001	<0.001	<0.001	<0.001	<0.001	<0.001

### 3.4 Logistic regression analysis of influencing factors for CAS in T2DM patients

This study now conducts a collinearity diagnosis of each variable. The results (**Supplementary Table 1**) show that all independent variables have VIFs less than 5, indicating that there is no serious multicollinearity problem among the independent variables. Whether patients with type 2 diabetes have carotid atherosclerosis as the dependent variable (Yes = 1, No = 0). The indicators that showed statistically significant differences in the univariate analysis were included as independent variables in the multivariate logistic regression analysis. The results (**Table 3**) showed that the duration of diabetes, HbA1c, LDL-C, Scr, and NETs are risk factors for carotid atherosclerosis in patients with type 2 diabetes (P<0.05). Among these, for every increase of 2.32 ng/mL (i.e., one standard deviation, SD) in NETs levels, the risk increased by 1.76-fold(*OR = 1.76, 95%CI 1.40–2.21*).

**Table 3:** Logistic regression analysis of influencing factors for CAS in patients with T2DM.

Variables	OriginalOR	SD	Standardized OR (per 1 SD)	95%CI	P value
Duration of DM	1.091	4.12	1.43	1.19–1.72	0.001
HbA1c	1.529	1.70	2.06	1.52–2.79	<0.001
LDL-C	1.672	0.52	1.31	1.11–1.37	0.002
Scr	1.016	10.3	1.18	1.03–1.33	0.013
NETs	1.275	2.32	1.76	1.40–2.21	<0.001

Standardized ORs were calculated as ORstd​=exp(β×SD).

### 3.5 ROC Curve for evaluating the predictive value of NETs in CAS among T2DM patients

**Figure 3:**
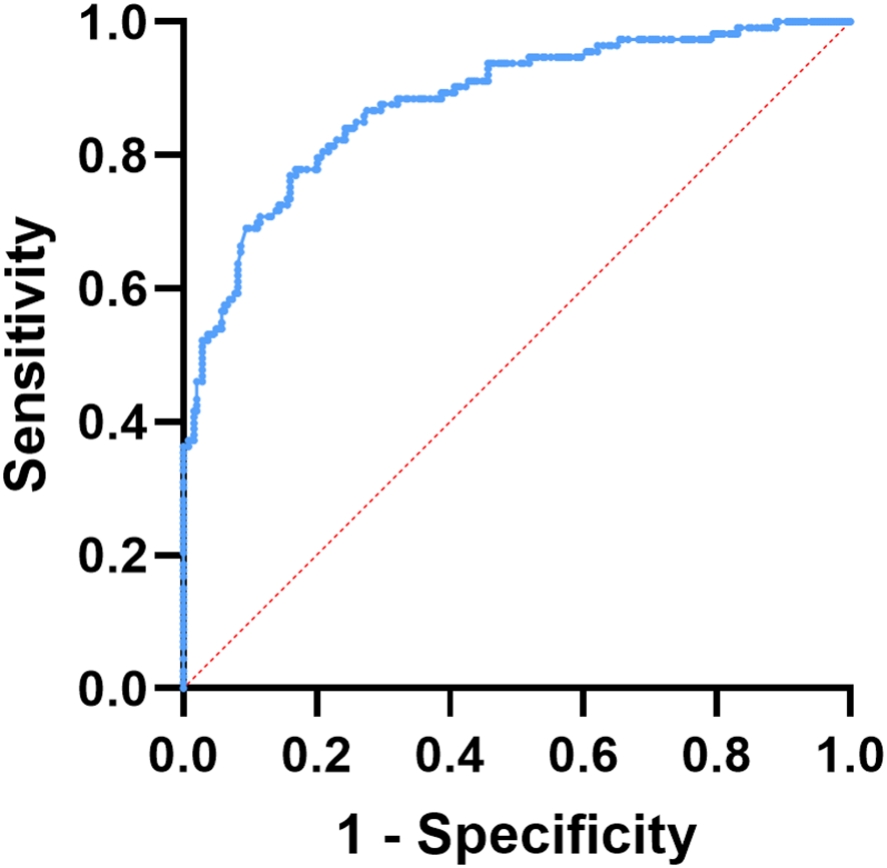
ROC curve of NETs for predicting CAS in patients with T2DM.

Receiver operating characteristic analysis was conducted to evaluate the ability of circulating NETs to discriminate increased carotid intima–media thickness and the presence of carotid plaque. The results demonstrated a strong diagnostic capacity, with an area under the ROC curve of 0.877 for serum NETs concentrations. When the threshold was set at 13.61, NETs achieved a sensitivity of 82.79% alongside a specificity of 77.69%, as illustrated in **Figure 3**.

## 4 Discussion

Atherosclerosis typically progresses asymptomatically in its early stages, making the carotid artery a critical window for observing atherosclerosis and assessing cardiovascular disease (CVD) risk (16). Evaluation of carotid atherosclerosis generally relies on two key approaches: one involves estimating plaque load through measurements of CIMT, while the other focuses on identifying the existence of atherosclerotic plaques directly (17). As a validated marker of subclinical atherosclerosis, CIMT has been shown to be very useful for cardiovascular disease risk assessment (18, 19). Elevated CIMT has been recognized as an independent indicator of cardiovascular risk and adverse events, and it shows a strong association with the development of macrovascular complications in individuals with type 2 diabetes mellitus (20–22).

Atherosclerosis is also a chronic low-grade inflammatory vascular disease (6). Growing evidence indicates that neutrophils are actively involved in the progression of atherosclerosis, with one possible mechanism involving NETosis (23). NETs directly induce endothelial dysfunction by activating endothelial cells, promoting atherosclerosis development as the initiating stage of atherosclerosis (6). In animal models, ApoE^−/−^ mice injected with lipopolysaccharide develop larger lesions due to the accumulation of bone marrow cells and NETs (24). NETs also accumulate abundantly in downstream vulnerable plaque regions (25). Peptidylarginine deiminase 4 (PAD4), which mediates histone citrullination, plays a crucial role in NET formation (26). Shimonaga et al. reported higher PAD4 levels in patients with unstable plaques compared to those with stable plaques (27). Both PAD4 knockout in bone marrow-derived cells and the use of deoxyribonuclease I significantly attenuated arterial intimal injury and plaque formation (28). The above indicates that NETs play a central role in the occurrence, development, and plaque destabilization of atherosclerosis. In this cross-sectional study, we further investigated the association of carotid atherosclerosis with biomarkers associated with NETs according to CIMT status grading. CIMT thickening was found to be significantly associated with inflammatory marker levels in addition to traditional risk factors such as age, smoking, blood pressure, lipid profile, and blood glucose. Patients diagnosed with CAS exhibited markedly elevated circulating levels of NETs compared with individuals who showed no evidence of the condition (*P < 0.001*). Our study also found that NETs levels were significantly positively correlated with CIMT and increased progressively with higher CIMT grades, which is consistent with the findings of Shimonaga et al. regarding the association between NETs and carotid plaque instability (29). Furthermore, this study extends this association to an early subclinical atherosclerosis marker (CIMT), confirming that NETs are abnormally elevated throughout the entire process of carotid atherosclerosis from intimal thickening to plaque formation. This suggests that NETs may be involved in the early onset and progression of CAS in patients with T2DM. Logistic regression analysis in this study confirmed that NETs are an independent risk factor for CAS in T2DM patients (*OR = 1.76, 95%CI 1.40–2.21, P < 0.001*). This corroborates findings from previous studies on the involvement of NETs in the inflammatory mechanisms of atherosclerosis (27). Moreover, by incorporating clinical characteristics of T2DM, this study further clarifies the synergistic effect of this risk factor with indicators such as diabetes duration, HbA1c, and LDL-C in the diabetic population. We performed ROC analysis to evaluate the predictive capability of NETs for CAS and found that NETs had a predictive value of 0.877 for CAS, with sensitivity and specificity of 82.79% and 77.69%, respectively, confirming their good predictive value for CAS in patients with T2DM. While existing studies have only confirmed the abnormal expression of NETs in patients with atherosclerosis (10), this study further defines the diagnostic threshold and efficacy in the specific diabetic population, providing a new potential biomarker for early screening of diabetic vascular complications in clinical practice. CIMT represents a structural marker of established subclinical atherosclerosis, reflecting long-term and cumulative vascular remodeling (30). In contrast, NETs are dynamic, inflammation-driven molecular mediators involved in the early stages of atherogenesis, which occur prior to overt intimal thickening (31). NETs reflect inflammatory activation and endothelial dysfunction in the vascular wall at an earlier stage, thereby enabling the identification of individuals at high risk but without evident structural vascular lesions. It may enable risk stratification at a pre-structural, potentially reversible stage. Increased NETs levels reflect a hyperinflammatory process characterized by excessive neutrophil activation, oxidative stress and endothelial injury, which cannot be fully represented by CIMT alone (32). Therefore, NETs may be used to identify a high inflammatory risk subgroup.Patients in this subgroup may have normal or only mildly elevated CIMT, but still present a significantly increased risk of atherosclerotic progression. These findings warrant validation in future large-scale prospective cohort studies, and interventional studies are needed to explore their potential as a therapeutic target.

AGEs were not included in the final regression model in this study, their univariate correlation with CIMT (r=0.150, P = 0.005) suggests that AGEs, as carriers of “metabolic memory,” may have significant interactions with NETs. From a pathophysiological perspective, the two may form a vicious cycle that collectively promotes vascular injury. On one hand, AGEs are potent inducers of NETs. Binding of AGEs to their receptor (RAGE) directly triggers NETosis by activating the generation of reactive oxygen species (ROS) in neutrophils (33, 34). This explains why patients with significantly increased CIMT often exhibit elevated levels of both AGEs and NETs. On the other hand, NETs can reciprocally amplify the damaging effects of AGEs. Proteases and oxidative products released by NETs not only directly damage the endothelium but may also upregulate RAGE expression on vascular cells, increasing tissue sensitivity to AGEs and thereby exacerbating the inflammatory response (7, 35). Once initiated, this “AGEs-NETs synergistic axis” may form a self-amplifying positive feedback loop. Hyperglycemia drives AGE accumulation, AGEs induce NET release, and NETs in turn enhance the pro-inflammatory effects of AGEs. This cycle may persist even after glycemic control is achieved (due to the metabolic memory effect of AGEs), leading to sustained vascular inflammation and ultimately driving progressive CIMT thickening. Therefore, future research should focus on validating the causal synergistic effects of these two factors in basic experiments, and exploring in clinical practice whether combined detection of AGEs and NETs can help more accurately identify high-risk populations.

Although this study employed a cross-sectional design and cannot definitively establish causality, integrating our data with the existing literature leads us to propose that NETs act as a driver, rather than a mere byproduct, in T2DM-associated atherosclerosis. Firstly, the elevation in NETs levels was observed in the early stages of CIMT thickening (the 1.0 mm ≤ CIMT < 1.5 mm group), rather than being present only after advanced plaque formation, which aligns with the temporal characteristics of a causative exposure. Secondly, the correlation of NETs with upstream metabolic stress indicators (HbA1c, AGEs) was stronger than with purely structural indices, suggesting that NETs may serve as a key mediator in the conversion of metabolic disturbances into vascular inflammation. Finally, a substantial body of basic research has mechanistically confirmed the pathogenic capacity of NETs to directly induce endothelial injury, promote lipid deposition, and amplify inflammation (6, 7, 35, 36). Therefore, NETs may represent not only a biomarker of diabetic vasculopathy but also a potential therapeutic target driving disease progression. However, this study cannot definitively confirm the direction of causality, and the role of “NETs as a driver” requires further validation through prospective and interventional studies.

This study has several limitations.In terms of study design and sample selection, the cross-sectional design does not allow the establishment of a causal relationship between serum NETs levels and carotid atherosclerosis in patients with type 2 diabetes. Second, this study only included a cohort of patients with type 2 diabetes; therefore, the findings require further validation before being generalized to other diabetic populations or healthy individuals. In addition, the limited sample size restricted our ability to fully account for potential confounding variables such as obesity in the analytical models.Regarding the detection method, serum NETs measured by ELISA only reflect the relatively stable fraction released into the circulation. This approach cannot capture the transient and dynamic process of NETosis, nor can it reflect NETs fragments rapidly degraded by endogenous nucleases. As a result, the measured NETs levels may underestimate the actual *in vivo* NETosis activity, and there may be a temporal discrepancy between the detection results and the pathophysiological processes of atherosclerosis development and progression, which affects the precise determination of the timing of NETs action.

Furthermore, regarding confounding factor control, we excluded patients who regularly used NSAIDs or systemic corticosteroids at the time of enrollment or within the four weeks prior to enrollment, as described in the Materials and Methods section. Although patients with regular use of these medications were excluded, we cannot completely rule out the potential impact of short-term, irregular use, or topical application of such preparations. These medications may influence NETosis levels or systemic inflammatory status. When constructing the multivariate logistic regression model, we selected covariates based on the following principles: Variables with P < 0.05 in univariate analysis; Traditional risk factors closely associated with CAS as confirmed by previous literature (such as age, gender, smoking, blood pressure, and blood lipids), which were included as potential confounders for adjustment even if they showed no statistical significance in univariate analysis in this study. We first performed collinearity diagnostics on all continuous independent variables, where a VIF > 5 was considered indicative of severe collinearity. The final model employed forward stepwise regression to identify factors independently associated with CAS. Additionally, we quantified the degree of association between NETs and each confounding factor using Spearman correlation analysis, providing a quantitative reference for assessing covariate weights and interpreting results in the regression model. Additionally, this study did not measure systemic inflammatory markers such as interleukin-6 (IL-6) and high-sensitivity C-reactive protein (hs-CRP). As core mediators of chronic inflammation in T2DM, these indicators are closely associated with NETs formation and vascular endothelial injury (6). The failure to include these markers in the analysis constitutes residual confounding bias, and we cannot completely rule out their indirect effects on the association between NETs and CAS. Therefore, the observed correlation between NETs and CIMT may, in part, reflect a broader inflammatory state.

In response to the above limitations, our future research directions are clearly defined as follows: first, to include a normoglycemic healthy control group to further verify the specificity of the association between serum NETs and carotid atherosclerosis in patients with type 2 diabetes; second, to conduct larger-scale longitudinal studies with long-term follow-up to clarify the causal relationship between the two, while expanding the sample size to improve the accuracy and robustness of the results and fully adjust for the effects of confounding variables such as obesity; third, to optimize detection methods by using a combination of more specific NETs biomarkers to accurately assess NETosis activity; fourth, to strengthen the refined control of confounding factors such as medication use, and minimize the interference of confounding factors on the study results through detailed collection of medication history and extended washout periods, so as to provide more conclusive evidence and insights for this field. Finally, future studies should incorporate a broader range of inflammatory markers into their models to elucidate whether NETs serve as an independent pathogenic factor or merely act as a surrogate marker of the overall inflammatory cascade.

## 5 Conclusion

Based on the findings of this study, elevated serum NETs levels are significantly and independently associated with increased CIMT in patients with T2DM, suggesting that neutrophil activation and NETs release may be closely related to the early stages of atherosclerosis. As an emerging inflammatory mediator, NETs may serve as a promising biomarker for assessing the risk of diabetic macrovascular complications. It should be emphasized that this study employed a cross-sectional design, which can only reveal associations between variables and cannot infer causality. Future large-scale prospective cohort studies and fundamental mechanistic experiments are warranted to elucidate the precise role of NETs in the pathogenesis and progression of diabetic vascular complications, as well as their potential as a therapeutic target.

## Data Availability

The original contributions presented in the study are included in the article/**Supplementary Material**. Further inquiries can be directed to the corresponding authors.

## References

[1] Einarson TR Acs A Ludwig C Panton UH . Prevalence of cardiovascular disease in type 2 diabetes: a systematic literature review of scientific evidence from across the world in 2007-2017. Cardiovasc Diabetol. (2018) 17:83. doi: 10.1186/s12933-018-0728-6, PMID: 29884191 PMC5994068

[2] Bos D Arshi B van den Bouwhuijsen QJA Ikram MK Selwaness M Vernooij MW . Atherosclerotic carotid plaque composition and incident stroke and coronary events. J Am Coll Cardiol. (2021) 77:1426–35. doi: 10.1016/j.jacc.2021.01.038, PMID: 33736825

[3] Bhattacharya P Kanagasooriyan R Subramanian M . Tackling inflammation in atherosclerosis: Are we there yet and what lies beyond? Curr Opin Pharmacol. (2022) 66:102283. doi: 10.1016/j.coph.2022.102283, PMID: 36037627

[4] Burgener SS Schroder K . Neutrophil extracellular traps in host defense. Cold Spring Harb Perspect Biol. (2020) 12:a037028. doi: 10.1101/cshperspect.a037028, PMID: 31767647 PMC7328462

[5] Brinkmann V Reichard U Goosmann C Fauler B Uhlemann Y Weiss DS . Neutrophil extracellular traps kill bacteria. Science. (2004) 303:1532–5. doi: 10.1126/science.1092385, PMID: 15001782

[6] Zhu Y Xia X He Q Xiao Q-A Wang D Huang M . Diabetes-associated neutrophil NETosis: pathogenesis and interventional target of diabetic complications. Front Endocrinol (Lausanne). (2023) 14:1202463. doi: 10.3389/fendo.2023.1202463, PMID: 37600700 PMC10435749

[7] Zheng F Ma L Li X Wang Z Gao R Peng C . Neutrophil extracellular traps induce glomerular endothelial cell dysfunction and pyroptosis in diabetic kidney disease. Diabetes. (2022) 71:2739–50. doi: 10.2337/db22-0153, PMID: 36095260

[8] Thakur M Junho CVC Bernhard SM Schindewolf M Noels H Döring Y . NETs-induced thrombosis impacts on cardiovascular and chronic kidney disease. Circ Res. (2023) 132:933–49. doi: 10.1161/CIRCRESAHA.123.321750, PMID: 37053273 PMC10377271

[9] Maruhashi T Higashi Y . Pathophysiological association between diabetes mellitus and endothelial dysfunction. Antioxidants (Basel). (2021) 10:1306. doi: 10.3390/antiox10081306, PMID: 34439553 PMC8389282

[10] Pieterse E Rother N Garsen M Hofstra JM Satchell SC Hoffmann M . Neutrophil extracellular traps drive endothelial-to-mesenchymal transition. Arterioscler Thromb Vasc Biol. (2017) 37:1371–9. doi: 10.1161/ATVBAHA.117.309002, PMID: 28495931

[11] Beckman JA Creager MA Libby P . Diabetes and atherosclerosis: epidemiology, pathophysiology, and management. JAMA. (2002) 287:2570–81. doi: 10.1001/jama.287.19.2570, PMID: 12020339

[12] Arnett DK Blumenthal RS Albert MA Buroker AB Goldberger ZD Hahn EJ . 2019 ACC/AHA guideline on the primary prevention of cardiovascular disease: A report of the American college of cardiology/American heart association task force on clinical practice guidelines. Circulation. (2019) 140:e596–646. doi: 10.1161/CIR.0000000000000678, PMID: 30879355 PMC7734661

[13] Paneni F . 2013 ESC/EASD guidelines on the management of diabetes and cardiovascular disease: established knowledge and evidence gaps. Diabetes Vasc Dis Res. (2014) 11:5–10. doi: 10.1177/1479164113512859, PMID: 24254974

[14] Kessenbrock K Krumbholz M Schönermarck U Back W Gross WL Werb Z . Netting neutrophils in autoimmune small-vessel vasculitis. Nat Med. (2009) 15:623–5. doi: 10.1038/nm.1959, PMID: 19448636 PMC2760083

[15] Masuda S Nakazawa D Shida H Miyoshi A Kusunoki Y Tomaru U . NETosis markers: Quest for specific, objective, and quantitative markers. Clin Chim Acta. (2016) 459:89–93. doi: 10.1016/j.cca.2016.05.029, PMID: 27259468

[16] Yang S Shen H You Y Fu Z Guo S Zhang Y . Phenotype-specific associations of circulating adipokine levels with carotid atherosclerosis: a systematic review and meta-analysis. Int J Cardiol Cardiovasc Risk Prev. (2025) 27:200543. doi: 10.1016/j.ijcrp.2025.200543, PMID: 41323746 PMC12662000

[17] Li C Huang Q Zhuang Y Chen P Lin Y . Association between Metrnl and carotid atherosclerosis in patients with type 2 diabetes mellitus. Front Endocrinol. (2025) 15:1414508. doi: 10.3389/fendo.2024.1414508, PMID: 39845885 PMC11750675

[18] Gardener H Caunca MR Dong C Cheung YK Elkind MSV Sacco RL . Ultrasound markers of carotid atherosclerosis and cognition: the Northern Manhattan study. Stroke. (2017) 48:1855–61. doi: 10.1161/STROKEAHA.117.016921, PMID: 28630235 PMC5482565

[19] Hunt D Hemmingsen B Matzke A Varghese C Hammerich A Luciani S . The WHO Global Diabetes Compact: a new initiative to support people living with diabetes. Lancet Diabetes Endocrinol. (2021) 9:325–7. doi: 10.1016/S2213-8587(21)00111-X, PMID: 33862005

[20] Cardoso CRL Salles GC Leite NC Salles GF . Prognostic impact of carotid intima-media thickness and carotid plaques on the development of micro- and macrovascular complications in individuals with type 2 diabetes: the Rio de Janeiro type 2 diabetes cohort study. Cardiovasc Diabetol. (2019) 18:2. doi: 10.1186/s12933-019-0809-1, PMID: 30630491 PMC6327523

[21] Willeit P Tschiderer L Allara E Reuber K Seekircher L Gao L . Carotid intima-media thickness progression as surrogate marker for cardiovascular risk: meta-analysis of 119 clinical trials involving 100–667 patients. Circulation. (2020) 142:621–42. doi: 10.1161/CIRCULATIONAHA.120.046361, PMID: 32546049 PMC7115957

[22] Lorenz MW Price JF Robertson C Bots ML Polak JF Poppert H . Carotid intima-media thickness progression and risk of vascular events in people with diabetes: results from the PROG-IMT collaboration. Diabetes Care. (2015) 38:1921–9. doi: 10.2337/dc14-2732, PMID: 26180107 PMC4580609

[23] Drechsler M Megens RTA van Zandvoort M Weber C Soehnlein O . Hyperlipidemia-triggered neutrophilia promotes early atherosclerosis. Circulation. (2010) 122:1837–45. doi: 10.1161/CIRCULATIONAHA.110.961714, PMID: 20956207

[24] Geng S Zhang Y Lee C Li L . Novel reprogramming of neutrophils modulates inflammation resolution during atherosclerosis. Sci Adv. (2019) 5:eaav2309. doi: 10.1126/sciadv.aav2309, PMID: 30775441 PMC6365109

[25] da Silva RF Baptista D Roth A Miteva K Burger F Vuilleumier N . Anti-apolipoprotein A-1 IgG influences neutrophil extracellular trap content at distinct regions of human carotid plaques. Int J Mol Sci. (2020) 21:7721. doi: 10.3390/ijms21207721, PMID: 33086507 PMC7588926

[26] Liu X Arfman T Wichapong K Reutelingsperger CPM Voorberg J Nicolaes GAF . PAD4 takes charge during neutrophil activation: Impact of PAD4 mediated NET formation on immune-mediated disease. J Thromb Haemost. (2021) 19:1607–17. doi: 10.1111/jth.15313, PMID: 33773016 PMC8360066

[27] Shimonaga K Matsushige T Takahashi H Hashimoto Y Yoshiyama M Ono C . Peptidylarginine deiminase 4 as a possible biomarker of plaque instability in carotid artery stenosis. J Stroke Cerebrovascular Dis. (2021) 30:105816. doi: 10.1016/j.jstrokecerebrovasdis.2021.105816, PMID: 33906071

[28] Franck G Mawson TL Folco EJ Molinaro R Ruvkun V Engelbertsen D . Roles of PAD4 and NETosis in experimental atherosclerosis and arterial injury: implications for superficial erosion. Circ Res. (2018) 123:33–42. doi: 10.1161/CIRCRESAHA.117.312494, PMID: 29572206 PMC6014872

[29] Shimonaga K Matsushige T Takahashi H Hashimoto Y Yoshiyama M Kaneko M . Association of neutrophil extracellular traps with plaque instability in patient with carotid artery stenosis. Ann Vasc Surg. (2022) 85:284–91. doi: 10.1016/j.avsg.2022.02.023, PMID: 35276352

[30] Yılmaz A Çon E . Long-term atherogenic dyslipidaemia burden, rather than visit-to-visit variability, is associated with carotid intima-media thickness. Biomedicines. (2026) 14:226. doi: 10.3390/biomedicines14010226, PMID: 41595759 PMC12839352

[31] Chistiakov DA Bobryshev YV Orekhov AN . Neutrophil’s weapons in atherosclerosis. Exp Mol Pathol. (2015) 99:663–71. doi: 10.1016/j.yexmp.2015.11.011, PMID: 26551083

[32] Yang Z Xiong M Tang X Wang P Cui J Chen Y . Ginsenoside Rb1 mitigates atherosclerosis in part through modulating FTO-mediated m6A RNA modification in NETs-induced endothelial activation. Front Pharmacol. (2025) 16:1631076. doi: 10.3389/fphar.2025.1631076, PMID: 40771928 PMC12325202

[33] Bansal S Siddarth M Chawla D Banerjee BD Madhu SV Tripathi AK . Advanced glycation end products enhance reactive oxygen and nitrogen species generation in neutrophils. vitro. Mol Cell Biochem. (2012) 361:289–96. doi: 10.1007/s11010-011-1114-9, PMID: 22048812

[34] Cooney R Ahmadi L Petreaca M . Role of advanced glycation end products in neutrophil extracellular trap formation. FASEB J. (2019) 33:542.9. doi: 10.1096/fasebj.2019.33.1_supplement.542.9, PMID: 33414642

[35] Mao J Wu S Yan Z Huang G Yu Y . Neutrophil extracellular traps as therapeutics target in vascular aging. Front Immunol. (2025) 16:1657938. doi: 10.3389/fimmu.2025.1657938, PMID: 41132659 PMC12540153

[36] Döring Y Soehnlein O Weber C . Neutrophil extracellular traps in atherosclerosis and atherothrombosis. Circ Res. (2017) 120:736–43. doi: 10.1161/CIRCRESAHA.116.309692, PMID: 28209798

