# Supplementary material for: Association between serum neutrophil extracellular traps and carotid intima-media thickness in type 2 diabetes: a cross-sectional study
Source: Front Endocrinol (Lausanne). 2026 Mar 17;17:1769035. doi: 10.3389/fendo.2026.1769035 (PMC13035717; doi:10.3389/fendo.2026.1769035)
Supplement: Supplementary file 1 [file Table1.docx]

**Supplementary materials**

**Table S1. Collinearity Statistics**

|  | **Unstandardized Coefficients** | | **Standardized Coefficients** |  |  | **Collinearity Statistics** | |
| --- | --- | --- | --- | --- | --- | --- | --- |
|  | B | Std. Error | Beta | t | Sig. | Tolerance | VIF |
| (Constant) | -1.287 | 0.284 |  | -4.540 | <.001 |  |  |
| Age | -0.003 | 0.002 | -0.045 | -1.219 | 0.224 | 0.803 | 1.246 |
| BMI | 0.013 | 0.006 | 0.083 | 2.411 | 0.016 | 0.935 | 1.070 |
| Gender | 0.122 | 0.046 | 0.099 | 2.650 | 0.008 | 0.806 | 1.241 |
| Tobacco Use History | 0.120 | 0.053 | 0.079 | 2.263 | 0.024 | 0.907 | 1.103 |
| Hypertension | 0.006 | 0.044 | 0.004 | 0.126 | 0.900 | 0.880 | 1.136 |
| AGE accumulation | 0.002 | 0.001 | 0.053 | 1.507 | 0.133 | 0.910 | 1.099 |
| FPG | -0.002 | 0.008 | -0.010 | -0.222 | 0.825 | 0.603 | 1.659 |
| 2hPG | 0.008 | 0.005 | 0.058 | 1.379 | 0.169 | 0.629 | 1.591 |
| HbA1c | 0.094 | 0.017 | 0.315 | 5.541 | <.001 | 0.346 | 2.892 |
| GA | 0.011 | 0.005 | 0.122 | 2.378 | 0.018 | 0.425 | 2.351 |
| TC | 0.003 | 0.006 | 0.018 | 0.492 | 0.623 | 0.872 | 1.147 |
| TG | 0.005 | 0.013 | 0.015 | 0.389 | 0.698 | 0.797 | 1.255 |
| HDLC | -0.040 | 0.081 | -0.018 | -0.499 | 0.618 | 0.818 | 1.222 |
| LDLC | 0.095 | 0.024 | 0.148 | 3.939 | <.001 | 0.793 | 1.261 |
| SCr | 0.001 | 0.001 | 0.057 | 1.600 | 0.111 | 0.880 | 1.136 |
| SUA | 0.000 | 0.000 | -0.063 | -1.756 | 0.080 | 0.857 | 1.166 |
| eGFR | 0.000 | 0.000 | 0.009 | 0.267 | 0.790 | 0.954 | 1.049 |
| NETs | .042 | 0.007 | 0.272 | 6.454 | <.001 | 0.631 | 1.585 |
| Duration of Diabetes | 0.015 | 0.003 | 0.170 | 4.753 | <.001 | 0.870 | 1.149 |

Dependent variable: CIMT
